# A novel age-biomarker-clinical history prognostic index for heart failure with reduced left ventricular ejection fraction

**DOI:** 10.1515/med-2020-0209

**Published:** 2020-07-10

**Authors:** Hao Li, Yuan Cui, Jin Tian, Hong Yang, Qing Zhang, Ke Wang, Qinghua Han, Yanbo Zhang

**Affiliations:** Department of Health Statistics, Shanxi Provincial Key Laboratory of Major Diseases Risk Assessment, School of Public Health, Shanxi Medical University, 56 South XinJian Road, Taiyuan, Shanxi Province 030001, People’s Republic of China; Department of Cardiology, First Hospital of Shanxi Medical University, 85 South XinJian Road, Taiyuan, Shanxi Province 030001, People’s Republic of China

**Keywords:** HFrEF, latent trait analysis, nomogram, NT-proBNP

## Abstract

**Purpose:**

A model for predicting the prognosis of patients with heart failure with reduced left ventricular ejection fraction (HFrEF) is currently not available. This study aimed to develop an age-biomarker-clinical history prognostic index (ABC-PI) and validate it for the assessment of individual prognosis.

**Patients and methods:**

A total of 5,974 HFrEF patients were enrolled and 1,529 were included in this study after excluding missing values and loss to follow-up. Variables that significantly contributed to prediction of all-cause mortality were assessed by Cox regression and latent trait analysis (LTA) was used to validate discrimination of variables.

**Results:**

After Cox regression, the following seven most significant variables were selected: age, N-terminal pro-B-type natriuretic peptide, renal dysfunction, left ventricular mass index, percutaneous coronary intervention, atrial fibrillation, and New York Heart Association (C-index: 0.801 ± 0.013). After verification by LTA, discrimination of these seven variables was proven. A nomogram was used to form the ABC-PI, and then the total score was set to 100 points. A lower score indicated a higher risk. After verification, the 3-year mortality rate was 34.7% in the high-risk group and only 2.6% in the low-risk group.

**Conclusion:**

Our novel ABC-PI shows a good performance and does not require re-input in the original model. The ABC-PI can be used to effectively and practically predict the prognosis of HFrEF patients.

## 1 Introduction

Approximately 26 million people suffer from heart failure worldwide, and it has become a global public health problem [1]. Heart failure is the most common cardiovascular cause of hospitalization in patients older than 60 years [2]. Heart failure has a high prevalence and mortality rate, and it can severely impair physical function and quality of life [3,4]. Heart failure with reduced left ventricular ejection fraction (HFrEF) is a complex condition. Patients with HFrEF may belong to different subpopulations and be associated with different risks of death. Therefore, creating an HFrEF prognosis model for determining the risk of death of these patients is desirable.

Previous studies on cardiovascular disease often focused on risk score systems to identify the risk of certain events rather than prognosis, such as using scores to predict the risk of bleeding in patients with atrial fibrillation [5]. Furthermore, in many classic risk scoring systems, biomarker variables, such as Framingham risk functions, are not included [6]. Measurement of biomarkers has become a routine examination during hospitalization, and it has a strong predictive power. Therefore, use of biomarkers should be considered when creating a prognostic index (PI). There are many studies exploring the biomarkers of cardiovascular disease [7,8]. A common effective biomarker in patients with HFrEF is N-terminal pro-B-type natriuretic peptide (NT-proBNP) [9]. Currently, the most commonly used indicator in patients with HFrEF is the New York Heart Association (NYHA) functional classification system, which was developed by the American College of Cardiology Foundation and the American Heart Association [10]. In this system, the NYHA classification is based on the patient’s symptoms and the degree to which the condition affects their daily activities. This classification also needs to be considered as an important variable for inclusion in a PI. Additionally, there are some other common predictors, such as atrial fibrillation [11].

Latent trait analysis (LTA), which is a form of latent structure analysis, was first proposed in 1968 [12]. LTA is used for measurement characteristics in different subpopulations, with educational testing and mental testing in particular. This method has been proven to be effective [13].

This study aimed to develop a new age-biomarker-clinical history prognostic index (ABC-PI) and to validate it for predicting the prognosis of patients with HFrEF. Discrimination of the ABC-PI was tested by LTA. This study established a PI using a nomogram that was expected not to require re-input of the original model to achieve the purpose of improving practicability and effectiveness.

## 2 Methods

### 2.1 Study population

Patients with HFrEF were enrolled at the First Hospital of Shanxi Medical University and Shanxi Cardiovascular Hospital. The cohort was created in January 2014, and the cutoff date for analyses was August 2019. The inclusion criteria were an age of ≥18 years; diagnosis of HFrEF (presence of a basic cardiovascular disease, such as coronary heart disease and hypertension, with typical symptoms of chronic heart failure, such as paroxysmal dyspnea, tiredness, palpitation, pulmonary rales, and pleural effusion); an ejection fraction of <40% or 40–50% with structural heart disease or diastolic dysfunction; and NYHA grade II, III, or IV. The exclusion criteria were the presence of a mental disease or another serious disease (e.g., malignant tumors). Patients had data recorded at baseline and were followed up via cell phone and annual visits for 3 years. After each patient was discharged from the hospital, they were followed up via cell phone (the phone number was recorded on their first admission to the hospital) to confirm survival status, and in cases of death, the exact date of death was recorded.

A total of 5,974 patients were initially included. A total of 1,529 patients were finally included in the study after excluding incomplete variable data and loss to follow-up. The patients were divided into two groups, including one for development of the PI (*n* = 878) and another for verification of the PI (*n* = 651).

### 2.2 HFrEF-electronic Case Report Form

An HFrEF-electronic Case Report Form (HFrEF-eCRF) was established to collect baseline information during hospitalization, which include the following: (1) basic information, such as demographic characteristics, admission time, discharge time, and clinical diagnosis; (2) comorbidities and history of allergies; (3) results of a standard physical examination; (4) laboratory test results; (5) results of imaging, including electrocardiography and echocardiography; and (6) medication and surgery information. A total of 222 variables were included in this study.

The HFrEF-eCRF was developed on the basis of clinical guidelines and clinicians’ opinions. Entry and follow-up personnel were trained, and data quality was controlled using dual entry. The study was reviewed and approved by the Medical Ethics Committee of Shanxi Medical University, China (No. 2013099), and written informed consent was obtained from all participants.

### 2.3 Statistical analysis

Mean values and standard deviations were calculated for continuous data, and numbers and percentages were calculated for categorical data. In the development stage, univariable and multivariate logistic regression was used to initially screen the variables. To improve predictive efficiency, the selected continuous variables were transformed into categorical variables via receiver operating characteristic (ROC) curve segmentation and re-introduced into model validation. A log-rank procedure was then used to verify the variables and the validated variables were included in Cox stepwise regression analysis.

LTA was used to test discrimination of selected variables. LTA is also called item response theory in the field of psychometrics. This method establishes a functional relationship between latent categories and external variables. To date, there are at least 20 types of latent trait models. According to different data, different models can be used to estimate the parameters. In our study, the most common Rasch model and Ltm were used. The Rasch model is a special case of a one-dimensional latent trait model and the Ltm is a two-parameter logistic model [14]. Parameter estimation of latent trait models generally uses maximum likelihood estimators [15]. The expectation–maximization algorithm and quasi-Newton algorithm are commonly used in the iterative process. In this study, the mixed algorithm was used to estimate parameters. Therefore, the expectation–maximization algorithm was used to iterate at the beginning, and then quasi-Newton algorithm was used to iterate until convergence. Evaluation methods of a model include the likelihood ratio test, Pearson’s test, the Akaike information criterion index, and the Bayesian information criterion index. Smaller values of the Akaike information criterion and Bayesian information criterion represent a better fit of the model [16,17,18]. In this study, the Akaike information criterion, Bayesian information criterion, and likelihood ratio test were used to compare model fitting. Additionally, the two-way residual was used to judge whether the model fit well. After the optimal model was determined, the observation value was substituted into the model to obtain the prediction value of the individual latent trait score. By comparing the latent trait scores of the death group and the survival group, we were able to verify whether the selected variables could divide patients with HFrEF into subpopulations (test discrimination of the selected variables).

Finally, the selected variables were input into a nomogram model. After a simple sum of the scores from the nomogram model, the ABC-PI was created [19] (univariate analysis: *α* = 0.05, multivariate analysis: *α*_entry_ = 0.05, and *α*_elimination_ = 0.10). All statistical analyses were performed using SPSS 26.0 (https://www.ibm.com/analytics/spss-statistics-software) and R 3.6.1 (https://www.r-project.org/) software.


**Ethics approval and informed consent:** The study was reviewed and approved by the Medical Ethics Committee of Shanxi Medical University, China (No. 2013099) and written informed consent was obtained from all participants.

## 3 Results

In the development group, diseases of these patients included coronary heart disease (388, 44.2%), old myocardial infarction (163, 18.6%), unstable angina pectoris (196, 22.3%), arrhythmia (83, 9.5%), and others (48, 5.5%). There were 195 (22.2%) deaths during the 3-year follow-up period. In the validation group, diseases of these patients included coronary heart disease (253, 38.9%), old myocardial infarction (147, 22.6%), unstable angina pectoris (113, 17.4%), arrhythmia (98, 15.1%), and others (40, 6.1%). There were 108 (16.6%) deaths during the 3-year follow-up period. The 222 baseline variables were tested one by one, and the standard basic variables and all variables that showed significant differences (*p* < 0.05) are shown in Table 1.

**Table 1:** Patients’ characteristics

Characteristic	*N*	Survival (*n* = 683)	Death (*n* = 195)	OR (95% CI)	*P*
Basic data					
Age (years)	878	67.91 ± 10.97	74.31 ± 9.92	1.065 (1.046–1.084)	<0.001
Sex					
Male	585	468 (80.0%)	117 (20.0%)		
Female	293	215 (73.4%)	78 (26.6%)	1.451 (1.044–2.016)	0.026
NYHA grade					
II	296	255 (86.1%)	41 (13.9%)		
III	357	288 (80.7%)	69 (19.3%)	1.490 (0.977–2.272)	0.064
IV	225	140 (62.2%)	85 (37.8%)	3.766 (2.466–5.781)	<0.001
Heart rate (per minute)	878	75.01 ± 16.07	79.95 ± 16.87	1.017 (1.008–1.027)	<0.001
Body mass index (kg/m^2^)	878	24.78 ± 3.49	23.49 ± 3.92	0.899 (0.856–0.943)	<0.001
Biochemical data					
Log NT-proBNP (ng/L)	878	3.08 ± 0.50	3.49 ± 0.45	5.978 (4.095–8.727)	<0.001
Hemoglobin (g/L)	878	136.61 ± 17.88	127.90 ± 21.98	0.976 (0.968–0.985)	<0.001
Albumin (g/L)	878	42.17 ± 4.88	39.87 ± 4.86	0.902 (0.869–0.935)	<0.001
γ-Glutamyl transpeptidase (U/L)	878	42.86 ± 54.47	52.84 ± 67.04	1.003 (1.000–1.005)	0.041
Creatinine (mmol/L)	878	90.83 ± 40.31	110.45 ± 66.32	1.008 (1.004–1.011)	<0.001
Other findings					
PCI					
−	629	468 (74.4%)	161 (25.6%)		
+	249	215 (86.3%)	34 (13.7%)	0.460 (0.307–0.688)	<0.001
Atrial fibrillation					
−	584	479 (82.0%)	105 (18.0%)		
+	294	204 (69.4%)	90 (30.6%)	2.013 (1.453–2.788)	<0.001
Renal dysfunction					
−	748	616 (82.4%)	132 (17.6%)		
+	130	67 (51.5%)	63 (48.5%)	4.388 (2.965–6.494)	<0.001
Mitral valve insufficiency					
−	47	39 (83.0%)	8 (17.0%)		
Mild	463	373 (80.6%)	90 (19.4%)	1.176 (0.531–2.604)	0.689
Moderate	290	222 (76.6%)	68 (23.4%)	1.493 (0.666–3.349)	0.331
Severe	78	49 (62.8%)	29 (37.2%)	2.885 (1.187–7.016)	0.019
Left ventricular mass index (g/m^2^)	878	123.89 ± 34.32	137.19 ± 45.25	1.009 (1.005–1.013)	<0.001

Abbreviations: OR, odds ratio; CI, confidence interval; PCI, percutaneous coronary intervention; NT-proBNP, N-terminal pro-B-type natriuretic peptide.

**Figure 1:**
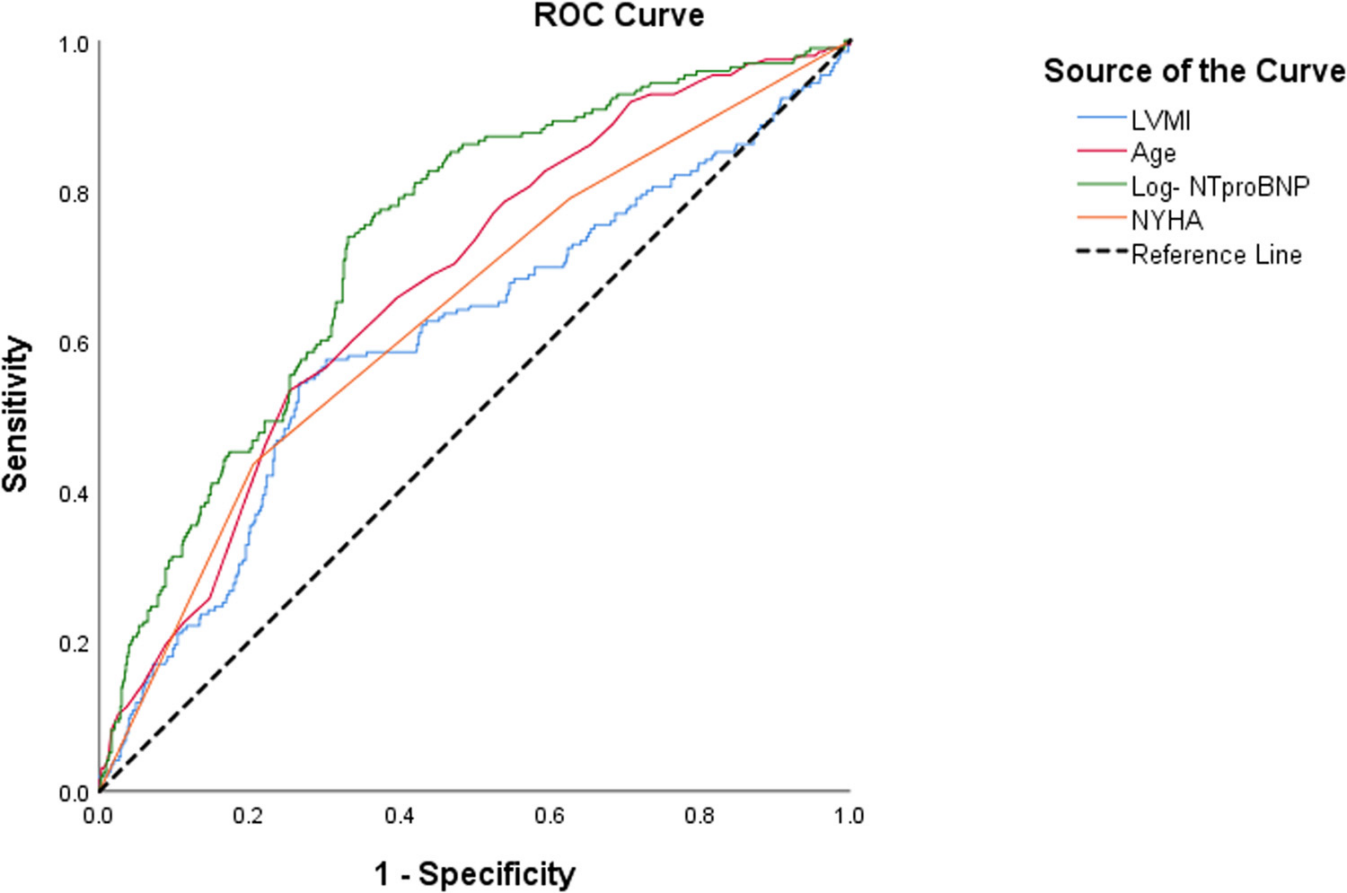
ROC curve. Abbreviations: ROC, receiver operating characteristic; LVMI, left ventricular mass index; NT-proBNP, N-terminal pro-B-type natriuretic peptide; NYHA, New York Heart Association.

Variables that were significantly different (*p* < 0.05) in Table 1 were included in multivariate logistic regression analysis. The overall predictive capacity of the resulting model was good (81.1%), and the predictive capacity was greater in the survival group (93.7%) than in the death group (36.9%). To improve the predictive capacity and clinical application value of the model, an ROC curve was used to identify the most sensitive cutoff value based on the results mentioned above (area under the curve ranged from 0.605 to 0.733; Figure 1). Additionally, continuous variables were converted into categorical variables. After conversion, the predictive capacity of the model was improved (81.9%). The effective prediction variables and cutoff value are shown in Table 2.

**Table 2:** Multivariate logistic regression results after conversion of variables

Variable	Cutoff value	*β*	SE	Wald *χ* ^2^	OR (95% CI)	*P*
Renal dysfunction		1.628	0.235	47.827	5.096 (3.212–8.085)	<0.001
PCI		−0.664	0.239	7.718	0.515 (0.322–0.822)	0.005
Atrial fibrillation		0.569	0.195	8.557	1.767 (1.207–2.588)	0.003
Log-NTproBNP (ng/L)	3.3268 (2122)	1.335	0.202	43.567	3.801 (2.557–5.650)	<0.001
NYHA grade	IV	0.664	0.202	10.767	1.943 (1.307–2.890)	0.001
Age	77	0.943	0.196	23.142	2.567 (1.748–3.769)	<0.001
LVMI	138	0.916	0.194	22.279	2.500 (1.709–3.658)	<0.001
Constant		−3.289	0.233	199.251	5.096 (3.212–8.085)	<0.001

Abbreviations: PCI, percutaneous coronary intervention; NT-proBNP, N-terminal pro-B-type natriuretic peptide; NYHA, New York Heart Association; LVMI, left ventricular mass index.

In the log-rank univariable test, all variables that showed significance in the logistic regression model passed the test. Multivariate Cox regression analysis was then performed. We found that not having had percutaneous coronary intervention (PCI), NT-proBNP levels >2,122 ng/L, a left ventricular mass index >137.9 g/m^2^, renal insufficiency, age >76.5 years, NYHA grade IV, and atrial fibrillation were risk factors for death in patients with HFrEF. The model’s prediction accuracy was high (*C*-index = 0.801 ± 0.013; Table 3).

**Table 3:** Multivariate Cox regression

Variable	*β*	SE	Wald *χ* ^2^	HR (95% CI)	*P*
PCI	−0.526	0.190	7.628	0.591 (0.407–0.858)	0.006
Log-NTproBNP (ng/L)	1.120	0.173	41.904	3.066 (2.184–4.304)	<0.001
LVMI	0.737	0.148	24.668	2.090 (1.563–2.797)	<0.001
Renal dysfunction	1.022	0.155	43.302	2.780 (2.050–3.769)	<0.001
Age	0.582	0.150	14.976	1.789 (1.332–2.401)	<0.001
NYHA grade	0.439	0.150	8.527	1.551 (1.155–2.082)	0.003
Atrial fibrillation	0.426	0.147	8.424	1.532 (1.149–2.043)	0.004

Notes: *C*-index: 0.801 ± 0.013.

Abbreviations: PCI, percutaneous coronary intervention; LVMI, left ventricular mass index; NT-proBNP, N-terminal pro-B-type natriuretic peptide; NYHA, New York Heart Association.

**Figure 2:**
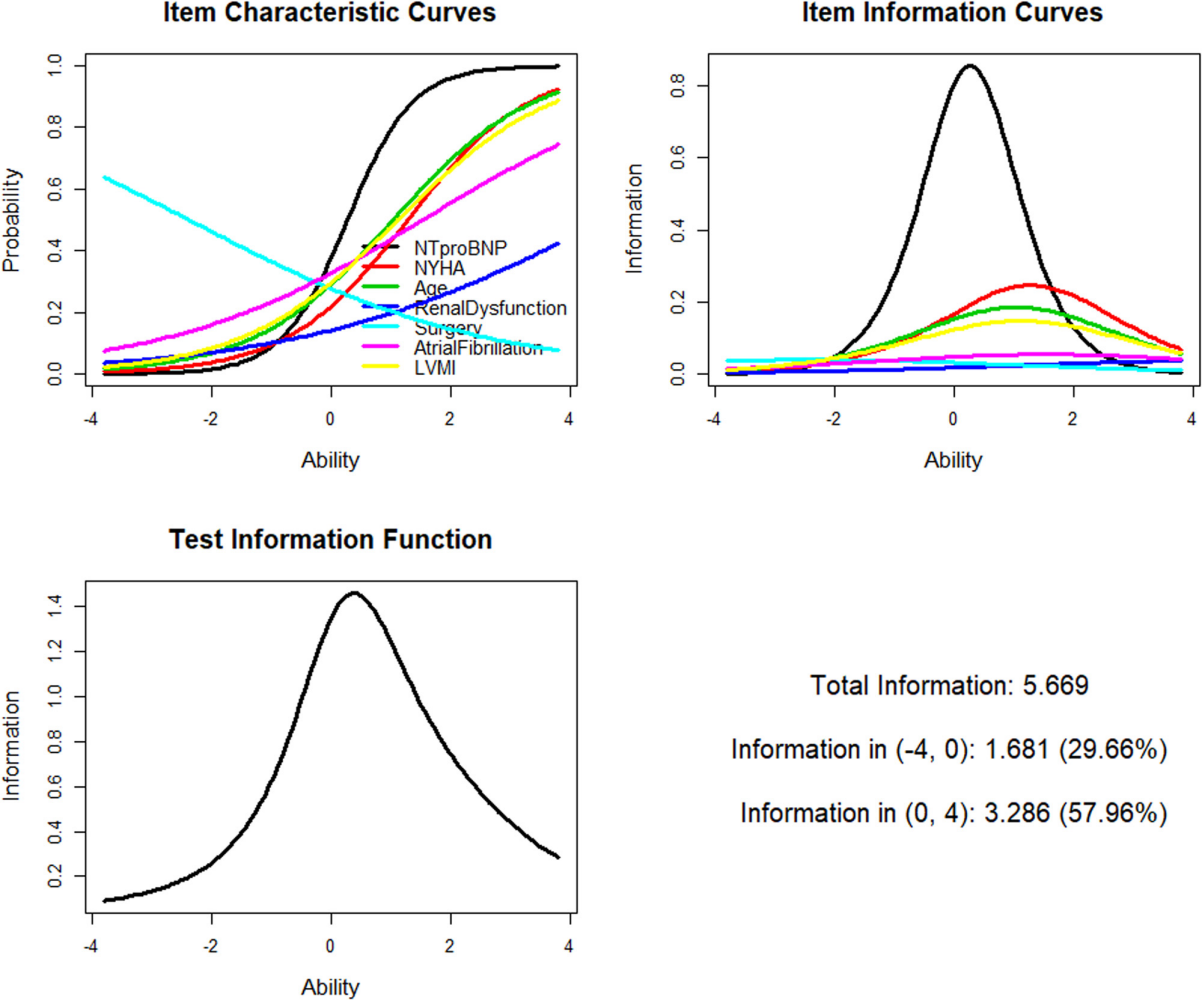
LTA curve. Abbreviations: LTA, latent trait analysis; LVMI, left ventricular mass index; NT-proBNP, N-terminal pro-B-type natriuretic peptide; NYHA, New York Heart Association.

LTA was used to verify whether the selected variables could effectively distinguish the latent trait of HFrEF in patients and data were used from the validation group. Figure 2 shows the parameter estimation results of LTA, and Table 4 shows comparison of the two LTA models. The Ltm was better than the Rasch model. The Ltm was used to fit the latent trait scores and the latent trait score results were converted to a 10-point system. The scores of the survival and death groups were compared by the *t*-test. In the death group, the mean latent trait score was 5.847 ± 2.055 and that in the survival group was 3.086 ± 2.228 (*t* = 16.233, *p* < 0.001). This finding indicated that the selected seven variables with great discrimination could effectively distinguish the latent trait of HFrEF in patients.

**Table 4:** Comparison of Ltm and Rasch model fitting indices in LTA

Model	AIC	BIC	Log.Lik	LRT	df	*p* value
Rasch	7319.76	7353.20	−3652.88			
Ltm	7253.80	7292.02	−3618.90	67.96	1	<0.001

Abbreviations: AIC, akaike information criterion; BIC, Bayesian information criterion, LRT, likelihood ratio test; df, degree of freedom.

**Figure 3:**
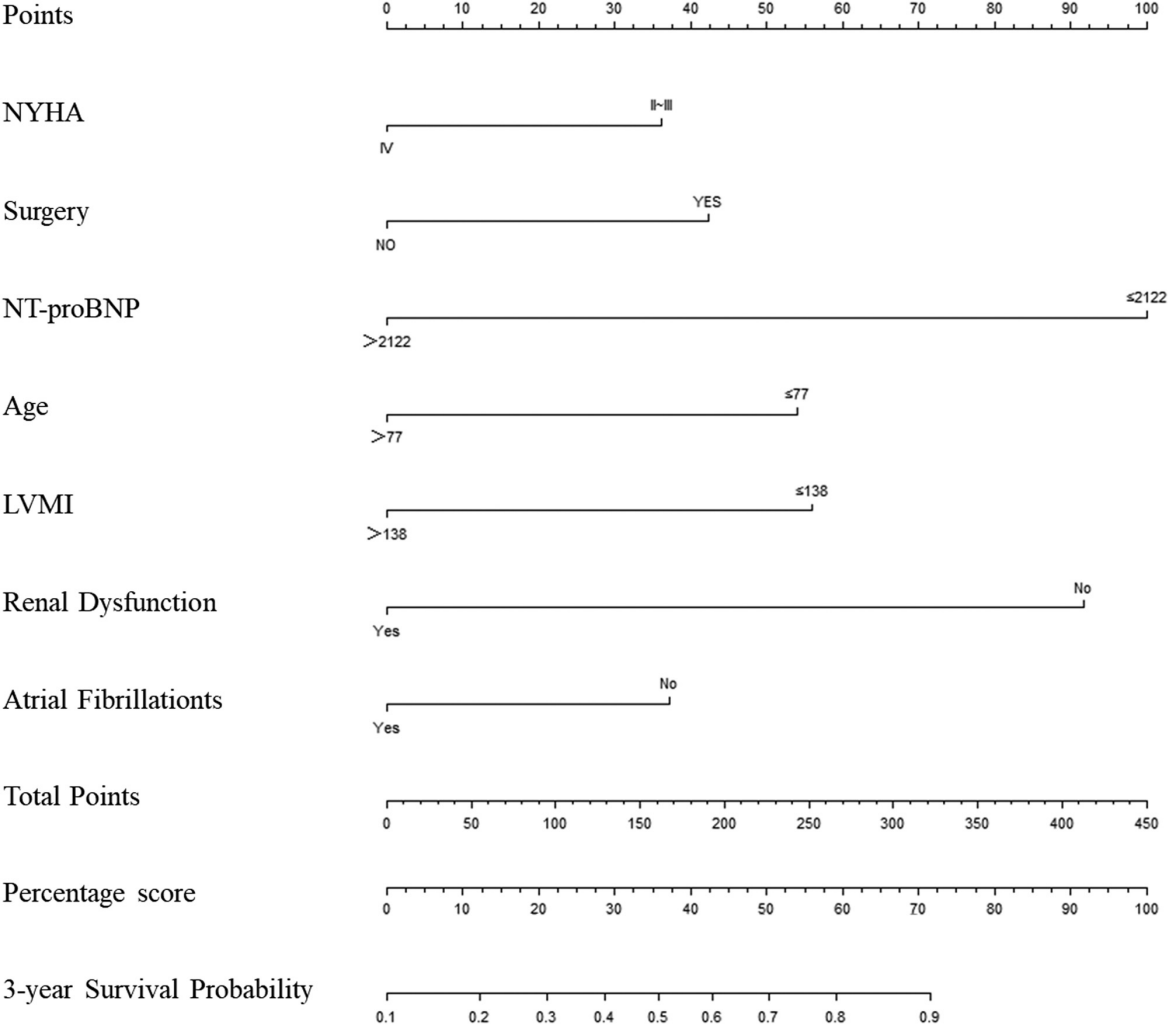
Nomogram. Abbreviations: NYHA, New York Heart Association; NT-proBNP, N-terminal pro-B-type natriuretic peptide; LVMI, left ventricular mass index.

Nomogram scores were examined using R software and the highest total score was 415. For ease of application, nomogram scores were converted to percentiles. The 3-year survival rate was >42% in patients with total scores >30 points and it was >82% in those with total scores >60 points (Figure 3). Table 5 shows accumulation of the total score of 100 points. NT-proBNP levels had the highest predictive value for death with a score of 24. NYHA grade had a low predictive value, with 8 points (for better application, cutoff values of the variables were rounded down to the nearest integer).

**Table 5:** Nomogram scores (centesimal system)

Variable	Category	Score
NT-proBNP (ng/L)	≤2,100	24
	>2,100	0
Renal insufficiency	−	22
	+	0
Age (years)	≤77	13
	>77	0
LVMI (g/m^2^)	≤140	13
	>140	0
PCI	−	0
	+	10
Atrial fibrillation	−	10
	+	0
NYHA grade	II/III	8
	IV	0
Total		100

Abbreviations: LVMI, left ventricular mass index; NT-proBNP, N-terminal pro-B-type natriuretic peptide; PCI, percutaneous coronary intervention; NYHA, New York Heart Association.

**Figure 4:**
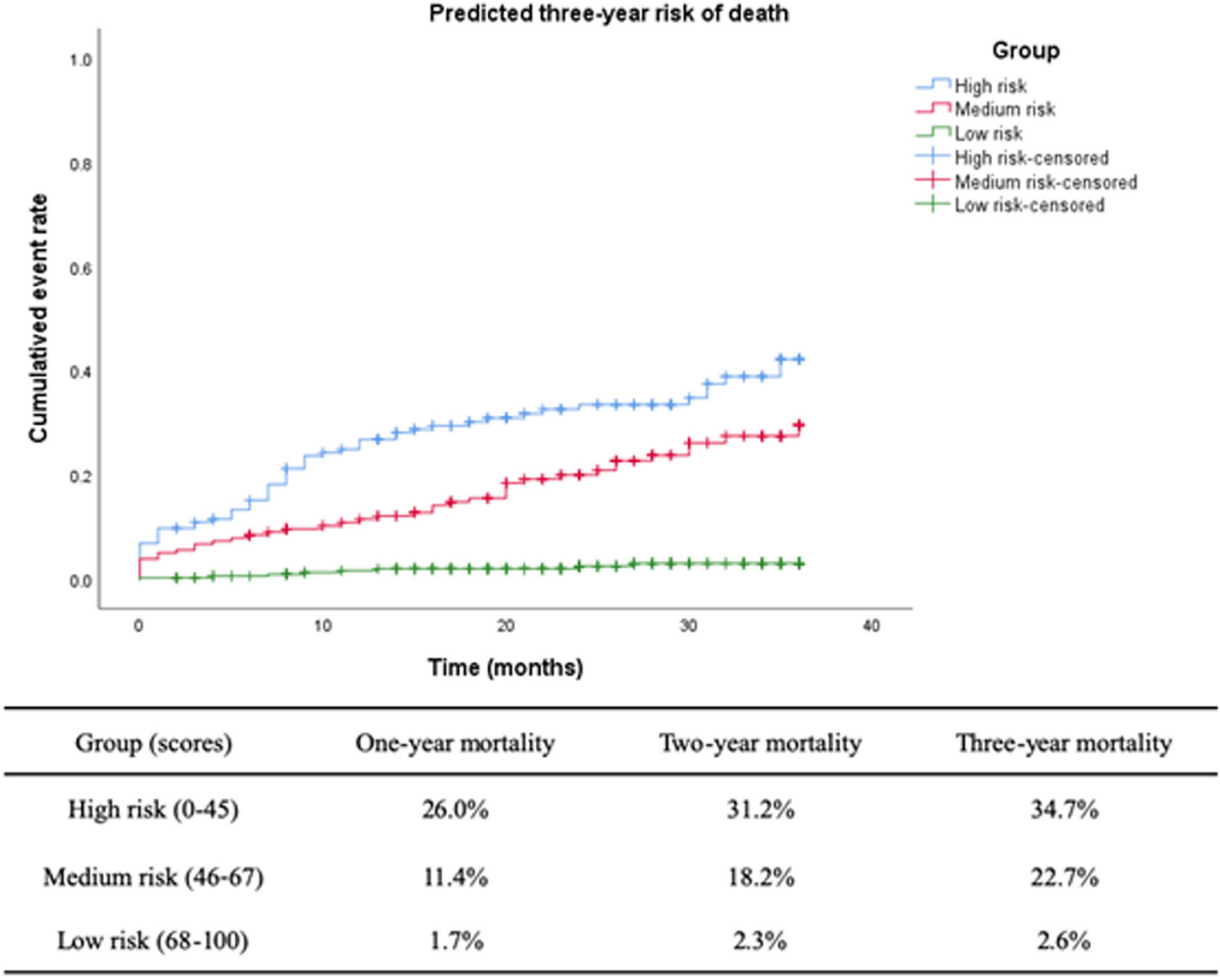
Cumulative risk of death predicted by the ABC-PI for different groups (validation group, *n* = 651). Abbreviations: ABC-PI, age-biomarker-clinical history prognostic index.

To further validate the ABC-PI, 651 patients in the validation group were tested. Higher scores were associated with lower 1-year, 2-year, and 3-year death rates (Figure 4). The 3-year mortality rate was 34.7% in patients with scores of ≤45, and it was only 2.6% in patients with scores of 68–100. The PI showed good performance.

## 4 Discussion

The prognosis of HFrEF differs substantially depending on subpopulations. Therefore, accurate assessment of this prognosis is important. In this study, we developed and validated the ABC-PI. The ABC-PI is a biomarker-based PI. The seven risk factors with the greatest capacity to predict risk of death in patients with HFrEF were NT-proBNP, renal dysfunction, age, left ventricular mass index, PCI, atrial fibrillation, and NYHA grade. After verification by LTA, the selected index was considered to be valid. The score was formed by a nomogram and then converted to a percentage system, and the ABC-PI was finally obtained. After external validation, the PI was found to be effective and practical.

The ABC indicator had a high accuracy because of inclusion of age, a biomarker, and clinical history. A good prognostic indicator can be used in many aspects, especially to evaluate the effectiveness of medicine and precise treatment. Hijazi et al. used the ABC score to determine the effectiveness of anticoagulants in patients with atrial fibrillation [20]. The ABC-PI for patients with HFrEF has not been determined yet. The ABC-PI used in our study could be useful for similar medical research.

The most informative predictor in the current study was NT-proBNP, which is a common biomarker of heart failure. Other biomarkers that have been identified include ST2 and galectin-3, but they are not routinely measured variables, and there is no evidence that their predictive capacity is better than that of NT-proBNP [21,22]. Therefore, NT-proBNP was used in the final PI in our study. Renal dysfunction is one of the most common comorbidities in patients with HFrEF. In a Swedish study, in patients with a reduced ejection fraction, those with renal dysfunction had a 1-year mortality rate of 23%, whereas those without renal dysfunction had a mortality rate of 8% [23]. In the previous study, after adjusting for other factors, the hazard ratio was 1.51 and the 95% confidence interval was 1.40–1.63. The hazard ratio of renal dysfunction in the present study was 2.78 (95% confidence interval: 2.05–3.77), which is similar to those of previous studies [24,25]. Age has a well-known important effect on the prognosis of HFrEF. A cutoff of 60 years was used when the variable of age was used to predict heart failure in some previous studies [25]. However, since the subsequent development of new medicines and surgical techniques, heart failure currently tends to progress more slowly. The life span of patients with heart failure has become prolonged, and accordingly, recent studies have determined that an age of 60 years is not an ideal cutoff. The best cutoff age may be between 67 and 80 years [26,27]. In the current study, with regard to the ROC curve, the optimal cutoff age was 77 years. This age may constitute a better cutoff for use in future studies. The left ventricular mass index is a combination of left ventricular mass and baseline body surface area. This index is associated with a variety of heart diseases and prognoses, including coronary heart disease, structural heart disease, and heart failure [28,29,30]. The left ventricular mass index also showed good predictive value for the prognosis of patients with HFrEF in the present study.

Traditionally, a PI that is generated on the basis of Cox regression is highly dependent on the model and requires re-input in the model. Based on the traditional Cox regression, this study used LTA to verify discrimination of the index. We then used a nomogram to form the ABC-PI, which did not depend on the original model, and it improved the practicability and applicability of the PI. LTA is commonly used in psychological tests and educational tests. LTA can distinguish different subpopulations of people [31]. Our study applied LTA to different latent traits with HFrEF, and it successfully validated discrimination of the selected variables. In the future, LTA could be used in similar diseases.

The current study has some limitations. The main limitation is that because the study population was limited to China, the results cannot necessarily be directly extrapolated to other populations. In future studies, we will attempt to incorporate heart failure research centers in other countries to further improve the accuracy of the ABC-PI.

## 5 Conclusion

Overall, the PI developed in the present study shows good performance. The variables that constitute the ABC-PI include age, biomarkers, and clinical history. Therefore, the scope of this index is comprehensive. After external validation, the ABC-PI performed well for predicting 1-year, 2-year, and 3-year mortality. The 1-year, 2-year, and 3-year mortality rates in the high-risk group were 26.0%, 31.2%, and 34.7% compared with 1.7%, 2.3%, and 2.6% in the low-risk group, respectively. In the future, the ABC-PI could be used for evaluating the effect of intervention and individual risk prediction.

## Abbreviations

ABC-PI: age-biomarker-clinical history prognostic index
eCRF: electronic Case Report Form
HFrEF: reduced left ventricular ejection fraction
LTA: latent trait analysis
NT-proBNP: N-terminal pro-B-type natriuretic peptide
NYHA: New York Heart Association
PCI: percutaneous coronary intervention
PI: prognostic index
ROC: receiver operating characteristic.
